# Clinical characteristics of patients with multi-canal benign paroxysmal positional vertigo

**DOI:** 10.1016/j.bjorl.2020.05.012

**Published:** 2020-06-16

**Authors:** Lihong Si, Xia Ling, Zheyuan Li, Kangzhi Li, Bo Shen, Xu Yang

**Affiliations:** Peking University Aerospace School of Clinical Medicine, Aerospace Center Hospital, Department of Neurology, Beijing, China

**Keywords:** Multiple-canals, BPPV, Nystagmus, Manual reduction

## Abstract

**Introduction:**

Multi-canal benign paroxysmal positional vertigo is considered to be a rare and controversial type in the new diagnostic guidelines of Bárány because the nystagmus is more complicated or atypical, which is worthy of further study.

**Objective:**

Based on the diagnostic criteria for multi-canal benign paroxysmal positional vertigo proposed by International Bárány Society, the study aimed to investigate the clinical characteristics, diagnosis and treatment of multi-canal benign paroxysmal positional vertigo.

**Methods:**

A total of 41 patients with multi-canal benign paroxysmal positional vertigo were included and diagnosed by Roll, Dix-Hallpike and straight head hanging tests. Manual reduction was performed according to the involvement of semicircular canals.

**Results:**

Among the 41 cases, 19 (46.3%) patients showed vertical up-beating nystagmus with or without torsional component and geotropic, apogeotropic horizontal nystagmus, and were diagnosed with posterior-horizontal canal. 11 (26.8%) patients showed vertical up-beating nystagmus with torsional component on one side and vertical down-beating nystagmus with or without torsional component on the other side during Dix-Hallpike test or straight head hanging test and were diagnosed with posterior-anterior canal benign paroxysmal positional vertigo 9 (26.8%) patients showed vertical down-beating nystagmus with or without torsional component and geotropic, apogeotropic horizontal nystagmus, and were diagnosed with anterior-horizontal canal 2 (4.9%) patients showed vertical geotropic torsional up-beating nystagmus on both sides and were diagnosed with bilateral posterior canal benign paroxysmal positional vertigo. High correlation between the sides with reduced vestibular function or hearing loss and the side affected by Multi-canal benign paroxysmal positional vertigo was revealed (contingency coefficient = 0.602, *p* = 0.010). During one-week follow up, nystagmus/vertigo has been significantly alleviated or disappeared in 87.8% (36/41) patients.

**Conclusion:**

Posterior-horizontal canal benign paroxysmal positional vertigo was the most common type. Multi-canal benign paroxysmal positional vertigo involving anterior canal was also not uncommon. Caloric tests and pure tone audiometry may help in the determination of the affected side. Manual reduction was effective in most of Multi-canal benign paroxysmal positional vertigo patients.

## Introduction

Benign paroxysmal positional vertigo (BPPV) is a paroxysmal, transient episode of vertigo triggered by specific head position changes; about 90% of positional vertigo is caused by BPPV, which is the most common peripheral vestibular disorder.^1^ The International Classification Committee of Vestibular Disorders of the Bárány Society published an expert consensus document on the diagnostic criteria for BPPV in the Journal of Vestibular Research in 2015.^2^ BPPV most often involves a single canal, which is called single canal BPPV (SC-BPPV) and can be classified according to the involved canals into three types: Posterior semicircular canal BPPV (PC-BPPV), lateral semicircular canal BPPV (LC-BPPV), anterior semicircular canal BPPV (AC-BPPV).^3^ Among the three types of BPPV, PC-BPPV and LC-BPPV are more common.^4^ PC-BPPV accounts for 60%–90% of all BPPV cases, LC-BPPV accounts for 5–30% of all cases,^4^, ^5^ and AC-BPPV is rare.^6^ BPPV may also involve multiple canals, which is called multi-canal BPPV (MC-BPPV).^5^, ^7^ Due to the involvement of different canals on different sides in MC-BPPV patients, the forms of nystagmus are more complicated or atypical, which usually have both vertical and horizontal components. Therefore, accurate diagnosis and treatment of MC-BPPV is difficult. At present, very few studies have focused on MC-BPPV; there is a lack of epidemiological data concerning the actual incidence of MC-BPPV. The proportion of MC-BPPV of all BPPV cases reported in previous studies was different. Moon et al.^5^ concluded that MC-BPPV accounted for no more than 5% of patients with BPPV, while Balatsouras et al.^8^ found that MC-BPPV is not uncommon, accounting for about 20% of patients with BPPV, and the accurate diagnosis of MC-BPPV resulted in successful treatment comparable with SC-BPPV. Given the above background, the present study aimed to analyze the clinical features, diagnosis and treatment of patients with MC-BPPV.

## Methods

All patients signed informed consent before inclusion and this study was approved by the ethics committee of Aerospace Center Hospital, Peking University Aerospace School of Clinical Medicine, China (n° 20151206-YN-07).

### Subjects

Forty-one patients with MC-BPPV who were treated in the vertigo department of our hospital from January 2016 to December 2017 were continuously enrolled. All patients met the following diagnostic criteria for MC-BPPV proposed by International Bárány Society^9^: recurrent episodes of vertigo or dizziness often provoked by lying down or turning over while supine; coexistence of positional nystagmus due to canalolithiasis in multiple semicircular canals evoked by Dix-Hallpike Test (D-HT) and Roll Test (RT); no other vestibular diseases.

### Clinical data collection

The following data were collected from patients’ medical records: gender, age, current medical history (course of disease, symptoms during attack, duration of attack, factors induced the attacks, triggering factors, accompanying symptoms), past medical history (including hypertension, diabetes, hyperlipidemia, coronary heart disease, migraine, cerebral infarction, brain injury, Meniere's disease, vestibular neuronitis, otitis media, osteoporosis, sleep disorders), results of physical examination upon admission, including RT and D-HT, and Straight Head-Hanging (SHH) tests, and auxiliary examinations (eye movement examination, bithermal caloric test, MRI and CT of the brain).

### Diagnosis of MC-BPPV

All patients underwent supine RT, D-HT and SHH; patients’ nystagmus was recorded using a Videonystagmograph (VNG). (1) RT: The patient was placed in supine position with head inclined forward 30°, the head was then quickly rotated 90° to one side, while maintaining this position for 1 min to observe for the presence of vertigo and nystagmus. Then the patient was returned to the supine position, the head was quickly rotated 90° to the opposite side, the position was maintained for 1 min to observe again for the presence of vertigo and nystagmus; finally, the patient was returned to the supine position. (2) D-HT: The patient was placed in a sitting position on the examination bed with the head turned to one side by 45°, then patient was rapidly laid down with the head hanging 30° below horizontal over the edge of the bed, nystagmus in this position was observed and recorded until the nystagmus disappeared, and the patient was then quickly returned to the sitting position. The other side was detected in the same manner. (3) SHH: The patient was placed in a sitting position on the examination bed and then rapidly laid down with straight head-hanging position where head is lowered at least 30° below the bed. In this position, the nystagmus of patient were observed and recorded until the nystagmus disappeared, the patient was then quickly returned to the sitting position.

The diagnosis of BPPV was based on typical nystagmus and clinical manifestations of patients: (1) A PC-BPPV diagnosis was given if D-HT and/or SHH test induced vertical up-beating nystagmus with or without torsional component; the nystagmus direction often reversed upon sitting up. And if vertical up-beating nystagmus with torsional component was induced by D-HT and/or SHH test (the torsional direction of the upper pole of the eye toward the affected side). (2) A LC-BPPV diagnosis was given if geotropic horizontal nystagmus was induced by supine RT, the side of stronger nystagmus was the affected side, and if apogeotropic horizontal nystagmus was induced, the side of weaker nystagmus was the affected side. (3) A AC-BPPV diagnosis was given if vertical down-beating nystagmus with or without torsional component was induced by D-HT and/or SHH tests, the nystagmus often reversed when sitting up. And if vertical down-beating nystagmus with torsional component was induced by D-HT and/or SHH tests (the torsional direction of the upper pole of the eye toward the affected side); (4) A MC-BPPV diagnosis was given if the typical nystagmus induced by D-HT/SHH test and supine RT was in accordance with the manifestations of multiple semicircular canal involvement; (5) patients were classified as having cupulolithiasis if the duration of nystagmus lasted ≥1 min, whereas they were classified as having canalolithiasis if the duration of nystagmus lasted <1 min.

### Treatments

Methods for treating PC-BPPV: (1) Epley's maneuvers^10^: Epley's maneuvers are used to treat canalithiasis of the posterior semicircular canal, and is performed as follows: the patient is placed in a sitting position on the examination bed, and the examiner turns the patient's head 45° to the affected side with both hands holding the patient's head, and with the help of the examiner, patient is quickly laid down in the supine position with the head hanging 30° below the bed, and then the head is turned 90° to the healthy side, while maintaining the position of the head and body, and the body of patient is rotated 90° to the healthy side, and returned to seated position until the vertigo has been alleviated or disappeared. During the reduction process, each position should be held for a certain period of time (usually for at least 30 s) until the nystagmus or vertigo disappears. (2) Semont maneuver^11^: The Semont maneuver is used to treat cupulolithiasis of the posterior semicircular canal. More specifically, the patient was placed in a sitting position on the middle of the examination bed with the legs hanging down, the patient was then quickly moved into a side-lying position on the affected side, with the head turned to the healthy side by 45°, the head position was maintained until the vertigo and nystagmus have disappeared. The patient was quickly moved into a side-lying position on the healthy side with the nose 45° horizontally. The patient was slowly returned to the sitting position until the vertigo and the nystagmus disappeared.

Methods for treating AC-BPPV^12^: Yacovino maneuver is the preferred method for the treatment of canalithiasis of the anterior semicircular canal, which is performed as follows: the patient was placed in a sitting position on the examination beds, and rapidly laid down with the head hanging 30°–75° below the bed, after maintaining the position for 30 s, the patient's head was elevated until the chin touched the chest. After maintaining the position for 30 s, the patient was slowly brought to sitting position with head bent down. Patient was then returned to the starting sitting position until the vertigo and the nystagmus disappeared.

Methods for treating LC-BPPV: (1) Barbecue maneuver^13^: Barbecue maneuver is a method used to treat LC-BPPV-canalithiasis, which is performed as follows: the patient was laid in supine position with head tilted 30° forward, the head and whole body were rotated 90° to the healthy side, then a further 90° rotation of the head and whole body toward the healthy side was performed (the patient laid in a prone position); the head and whole body were then rotated 90° to the healthy side, and finally a further 90° rotation was performed and the patient was turned to supine position. Each position should be maintained for a certain period of time until the nystagmus or vertigo disappeared. During this procedure, the otolithic debris is allowed to move out of the lateral semicircular canal and back to the utricle.^14^ (2) Gufoni's maneuver^15^: Gufoni's maneuver is used to treat cupulolithiasis in the lateral semicircular canal. The patient was placed in a sitting position on the middle of the examination bed with the legs hanging down, then the patient was quickly moved into a side-lying position on the affected side, while maintaining the position for 1–2 min. After the vertigo and the nystagmus have alleviated or disappeared, the patient's head was quickly rotated 45° toward the floor, kept in this position for 1–2 min, then the patient was slowly returned to the sitting position after the vertigo disappeared.^14^

### Treatment outcomes and follow-up

The efficacy of manual reduction methods in treatment of BPPV was classified into three grades: (1) Ineffective: positional vertigo and nystagmus were not alleviated, which had been much worse or converted to another type of nystagmus; (2) Effective: positional vertigo or nystagmus were alleviated, but did not disappear; (3) Cure: positional vertigo or nystagmus disappeared completely.

The appropriate manual reduction method was selected according to the type of MC-BPPV. The treatment efficacy was evaluated by Videonystagmography (VNG) at 2 h after the repositioning maneuver. All patients were followed up one week later. For uncured patients, the RT, D-HT, SHH test and -repositioning maneuver were performed again. The treatment efficacy was also evaluated at 2 h after reduction.

### Statistical analysis

All quantitative data were represented as mean ± Standard Deviation (SD), and for the data in each group following a normal distribution, the independent sample *t*-test was used to compare means of two groups. Enumeration data were expressed as percentage, Chi-Square (*χ*^2^) test was used to compare the difference between groups, and if necessary, the Yates continuity correction or Fisher's exact test were performed. All tests were two-sided, *p*-value of <0.05 was considered statistically significant. All data were analyzed using software package SPSS20.0.

## Results

### Clinical baseline characteristics of the patients

**Figure 1:**
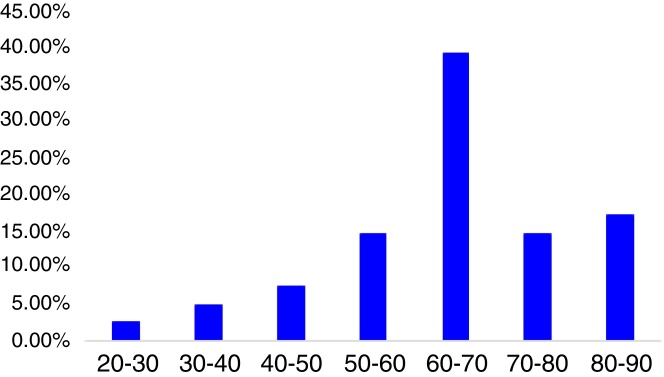
Proportion of patients with MC-BPPV in different ages.

A total of 41 patients with MC-BPPV were included in the study, accounting for 10.5% (41/396) of all BPPV patients visiting our department during the same period of time. There were 15 males (36.6%), 26 females (63.4%), with a women-to-men ratio of 1:1.73. The mean age of the patients was 64.2 ± 13.9 years (rang 27–87 years). The peak age for onset of MC-BPPV was between 61 and 70 years, accounting for 39.0% of all cases (Fig. 1).

### Clinical manifestations

At the time of disease onset, vertigo was present in 30 patients (73.2%), dizziness was present in 11 patients (26.8%). And among all patients, 11 patients (26.8%) presented with gait instability.

The duration of symptoms was 0–3 days in 21 patients (51.3%), 4–7 days in 8 patients (19.5%), 8–14 days in 6 patients (14.6%), 15–30 days in 3 patients (7.3%), 31–60 days in 3 patients (7.3%).

Among all patients with MC-BPPV, no obvious cause was found in 36 patients (87.8%); the causes of MC-BPPV were head trauma in 2 patients (4.9%), and preceding infection in 3 patients (7.3%). And concomitant factors were found to be fatigue in 2 patients (4.9%), sleep disturbance in 8 patients (19.5%).

### Patients’ medical history

Previous disease history included hypertension (*n* = 25, 60.1%), hyperlipidemia (*n* = 23, 56.1%), osteoporosis (*n* = 17, 41.5%), cervical spondylosis (*n* = 13, 31.7%), coronary heart disease (*n* = 9, 22.0%), diabetes (*n* = 10, 24.4%), migraine (*n* = 6, 14.6%), autoimmune diseases (*n* = 2, 4.9%), cerebral infarction (*n* = 3, 7.3%), sudden hearing loss (*n* = 3, 7.3%), brain trauma (*n* = 2, 4.9%), vestibular neuronitis (*n* = 3, 7.3%), and previous occurrences of BPPV (*n* = 8, 19.5%).

### The affected side of MC-BPPV

**Figure 2:**
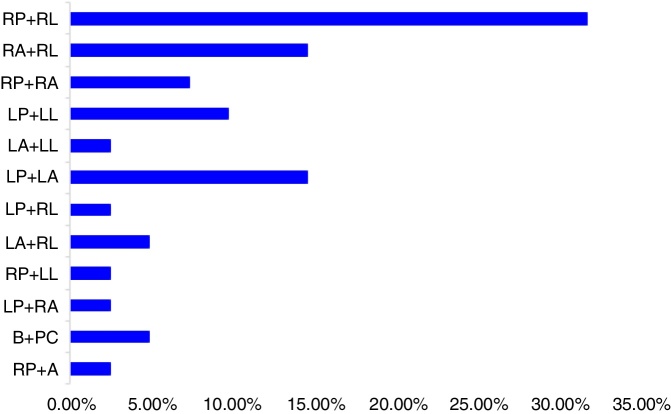
Distribution of side involvement of MC-BPPV.

One side was affected in 31 (75.6%) patients, both sides were affected in 9 (22.0%) patients, and the affected side was not identified in 1 (2.4%) patient. Among patients with one side affected, right side was affected in 21 (51.2%) patients, and the left side was affected in 10 (24.4%) patients (Fig. 2).

All 41 patients with MC-BPPV underwent bithermal caloric test, the results showed that 34 (82.9%) patients had normal vestibular function, and 7 (17.1%) patients had unilateral vestibular dysfunction, 3 (7.3%) of them experienced reduced vestibular function on right side, and 4 (9.8%) of them experienced reduced vestibular function on left side. And among these 7 patients, the sides with vestibular function reduction were consistent with the affected side of involved semicircular canal of BPPV in 5 (71.4%) patients, which was inconsistent in 2 (28.6%) patients.

Forty-one patients with MC-BPPV underwent pure tone audiometry. The results showed normal hearing in 32 (78.0%) patients, unilateral hearing loss in 8 (22.0%) patients, including 3 (7.3%) patients of hearing loss in the left ear, and 5 (12.2%) patients of hearing loss in the right ear, and 1 (2.5%) patient with bilateral hearing loss. Among those 8 patients, the sides with hearing loss were consistent with affected side of involved semicircular canal of BPPV in 6 (75.0%) patients, which were inconsistent in 2 (25.0%) patients.

The Fisher's exact test was used to further determine the correlation between the sides with reduced vestibular function or hearing decline and the affected side of MC-BPPV; the results revealed high correlation between the sides with reduced vestibular function or hearing decline and the side affected by MC-BPPV (contingency coefficient = 0.602, *p* = 0.010), and high concordance was observed by Kappa statistic (*K* = 0.647, *p* = 0.004).

### Types of MC-BPPV

According to the nystagmus characteristics, 19 patients (41.5%) were diagnosed as PC-LC-BPPV, as those patients showed both vertical torsional up-beating nystagmus and geotropic, apogeotropic horizontal nystagmus. Vertical up-beating nystagmus with torsional component on one side and vertical down-beating nystagmus with or without torsional component on the other side was induced by the Dix-Hallpike test in 11 patients (26.8%), and those patients were diagnosed with AC- PC-BPPV. Among these 11 patients with AC-PC-BPPV, up-beating nystagmus with a geotropic torsional component on one side in addition to down-beating nystagmus on the other side were induced by the D-H test in 8 patients; up-beating nystagmus with geotropic torsional component, lasting several dozen seconds, followed by down-beating nystagmus was induced by a D-H test on one side, and a down-beating nystagmus was induced by a D-H test on the other side in one patient; in one patient, up-beating nystagmus with geotropic torsional component, lasting several dozen seconds, followed by down-beating nystagmus, lasting ≥1 min was induced by a D-H test on one side, no nystagmus was induced by D-HT test on the other side, and down-beating nystagmus, lasting >1 min was induced during SHH test; and, down-beating nystagmus without torsional component was induced on both sides during bilateral D-HT and SHH tests in one patient, and the affected side cannot be determined.

Nine patients (26.8%) showed vertical down-beating nystagmus with or without torsional component and geotropic, apogeotropic horizontal nystagmus, and were diagnosed as AC-LC-BPPV; 2 patients (4.9%) developed vertical geotropic torsional up-beating nystagmus on both sides, and were diagnosed as bilateral PC-BPPV (Table 1, Table 2).

**Table 1:** Nystagmus characteristics in patients with LC-BPPVcombined PC- and AC-BPPV.

N°	Roll-test_L			Roll-test_R			Dix-Hallpike_L			Dix-Hallpike_R			CP	HL	Diagnose
	*n*	SPV(°/s)	ND(s)	*n*	SPV(°/s)	ND(s)	*n*	SPV(°/s)	ND(s)	*n*	SPV(°/s)	ND(s)			
1	AG	16	>60	AG	9	>60	DBN	11	45	–	–	–	R↓	–	RLC-cu + LAC
2	G	10	20	G	15	25	G	9	40	CCW + UBN	9	>60	–	–	RLC + RPC-cu
3	G	13	10	G	49	15	–	–	–	CCW + UBN	8	20	–	–	RLC + RPC
4	G	6	25	G	11	30	CW + UBN	29	40	G	11	15	–	–	RLC + LPC
5	G	64	25	G	30	20	G	11	>60	CCW + UBN	7 (HRBN) 11 (VUBN)	>60	–	–	LLC + RPC-cu
6	G	18	25	G	10	35				DBN	11	40	–	–	LLC + LAC
7	AG	18	>60	AG	12	>60	–	–	–	CCW + UBN	24	40	R↓	–	RLC-cu + RPC
8	AG	24	>60	AG	15	>60	–	–	–	CW + DBN	6	>60	–	–	RLC-cu + LAC-cu
9	G	10	15	G	24	30	–	–	–	CCW + UBN	16	>60	–	–	RLC + RPC-cu
10	G	12	20	G	25	30	–	–	–	CCW + UBN	10	15	–	–	RLC + RPC
11	G	10	20	G	28	25	–	–	–	CCW + UBN	9	20	–	–	RLC + RPC
12	AG	18	30	AG	10	20	–	–	–	CCW + UBN	12	20	–	–	Anterior arm of RLC-ca + RPC
13	G	25	15	G	31	20	CCW + DBN	9 (HRBN) 8 (VDBN)	10	–	–	–	–		RLC + RAC
14	AG	15	>60	AG	8	>60	DBN	18	>60				R↓	–	RLC-cu + RAC-cu
15	G	48	10	G	15	10	–	–	–	CCW + UBN	7 (HLBN) 9 (VUBN)	>60		–	LLC + RPC-cu
16	G	14	>60	G	20	>60	–	–	–	CCW + UBN	12	>60	–	R↓	RLC-cu + RPC-cu
17	G	19	20	G	52	25	CCW + DBN	21 (HLBN) 22 (VDBN)	>60	UBN	34°/s	>60	–	R↓	RLC + RAC-cu
18	G	12	15	G	31	25				CCW + UBN	13	10	–		RLC + RPC
19	AG	8	10	AG	20	15	CW + UBN	9	>60				–		Anterior arm of LLC-ca + RPC-cu
20	AG	10	>60	AG	17	>60	CW + UBN	8 (HLBN) 16 (VUBN)	>60				L↓		LLC-cu + LPC-cu
21	AG	15	10	AG	8	20				CW + DBN	11	15	–	–	Anterior arm of RLC-ca + LAC
22	AG	29	>60	AG	10	>60	CCW + DBN	22	>60	–	–	–	–		RLC-cu + RAC-cu
23	G	67	>60	G	27	>60				CCW + UBN	16	10	–		LLC-cu + RPC
24	AG	17	>60	AG	8	>60	DBN	13	15	DBN	22	25	–	R↓	RLC-cu + RAC
25	G	15	35	G	9	45	CW + UBN	9	15	–	–	–	–		LLC + LPC
26	G	18	35	G	43	35				CCW + UBN	16	10	–		RLC + RPC
27	G	10	15	G	19	20				CCW + UBN	28 (HLBN) 30 (VUBN)	15	L↓		RLC + RPC
28	AG	14	20	AG	6	15				CCW + UBN	28 (HLBN)	25	–	R↓	Anterior arm of RLC-ca + RPC

N, Nystagmus; SPV, Slow-Phase Velocity; ND, Nystagmus Duration; AG, Apogeotropic; G, Geotropic; s, seconds; HL, Hearing loss; DBN, Down-Beating Nystagmus; UBN, Up-Beating Nystagmus; R, Right; L, Left; CCW, Counterclockwise; CW, Clockwise; LAC, Left Anterior Canal; RAC, Right Anterior Canal; LPC, Left Posterior Canal; RPC, Right Posterior Canal; RLC, Right Lateral Canal; LLC, Left Lateral Canal; ca, canalolithiasis; cu, cupulolithiasis; HLBN, Horizontal Left-Beating Nystagmus; VUBN, Vertically Up-Beating Nystagmus; HRBN, Horizontal Right-Beating Nystagmus; VDBN, Vertically Down-Beating Nystagmus; G, beats toward the ground; ApoG, beats toward the ceiling.

**Table 2:** Nystagmus characteristics in patients with MC-BPPV involving the AC and PC.

N°	*n*	Dix-Hallpike_L	ND(s)	*n*	Dix-Hallpike_R	ND(s)	*n*	SHH	ND(s)	CP	HL	Diagnose
		SPV(°/s)			SPV(°/s)			SPV(°/s)				
1	DBN	6 (HLBN) 11 (VDBN)	>60	CCW + UBN-DBN	11 (HLBN) 22 (VUBN)	15	CCW + UBN-DBN	12 (HLBN) 18 (VUBN)	15	–	–	RPC + AC-unclear side–cu
					8 (DBN)	>60		11 (DBN)	>60			
2	CW + UBN	10	50	–	–	–	DBN	12	50	L↓	L↓	LPC + LAC
3	DBN	12	>60	CCW + UBN	40	15	DBN	12		–	R↓	RPC + RAC
4	CW + UBN-DBN	12 (UBN)	10	DBN	6	20	–	–		R↓	–	LPC + LAC
		11 (DBN)	20									
5	CW + UBN	12	30	DBN	15	40	DBN	20	40	–		LPC + LAC
6	CW + UBN	9	15	DBN	19	>60	DBN	19	>60	–	B↓	LPC + LAC-cu
7	DBN	10	20	CCW + UBN	16 (HLBN) 10 (VUBN)	10	DBN	8	10	–	R↓	RPC + RAC
8	CW + UBN	6	10	CCW + UBN	25	15	UBN	20	15	–		Bilateral PC-ca
9	CW + UBN	17	>60	DBN	10	>60				–	L↓	LPC-cu + LAC-cu
10	CW + UBN	15	20	CCW-UBN	25	25				–		Bilateral PC-ca
11	CW + UBN- CCW + DBN	20 (HRBN) 38 (VUBN)	10				CCW + DBN	8	>60	–	–	LPC + RAC-cu
		15 (DBN)	>60									
12	DBN	8	30	CW + UBN	9	25				–	L↓	RPC + RAC
13	CW + UBN	22	10	DBN	6	30				–	–	LPC + LAC

N, Nystagmus; SPV, Slow-Phase Velocity; ND, Nystagmus Duration; s, seconds; HL, Hearing Loss; DBN, Down-Beating Nystagmus; UBN, Up-Beating Nystagmus; R, Right; L, Left; CCW, Counterclockwise; CW, clockwise; LAC, Left Anterior Canal; RAC, Right Anterior Canal; LPC, Left Posterior Canal; RPC, Right Posterior Canal; RLC, Right Lateral Canal; LLC, Left Lateral Canal; ca, canalolithiasis; cu, cupulolithiasis; HLBN, Horizontal Left-Beating Nystagmus; VUBN, Vertically Up-Beating Nystagmus; HRBN, Horizontal Right-beating Nystagmus; VDBN, Vertically Down-Beating Nystagmus; G, beats toward the ground; ApoG, beats toward the ceiling; AC, Anterior cana; PC: posterior Canal.

**Figure 3:**
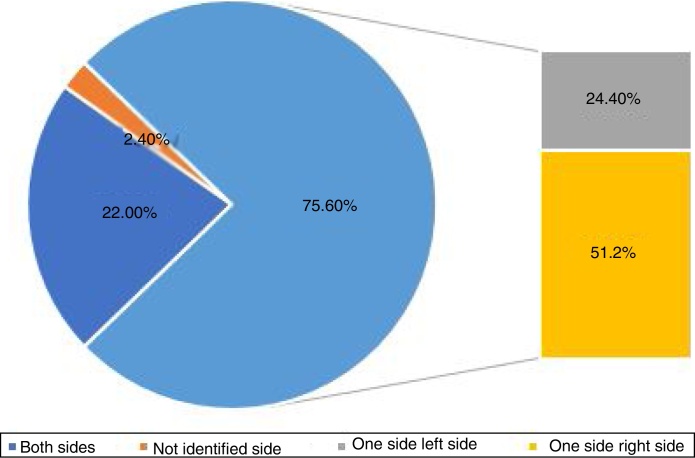
Distribution of different types of MC-BPPV.

According to the semicircular canals involved, the right PC combined with right LC were involved in 13 (31.7%) patients, the right AC combined with the right LC in 6 (14.6%) patients, the right PC combined with the right AC in 3 (7.3%) patients, the left PC combined with left LC in 4 (9.8%) patients, left AC combined with left LC in 1 (2.4%) patient, left PC combined with left AC in 6 (14.6%) patients, left PC combined with right LC in 1 (2.4%) patients, left AC combined with the right LC in 2 (4.9%) patients, the right PC combined with the left LC in 1 (2.4%) patient, the left PC combined with the right AC in 1 (2.4%) patient, bilateral PC in 2 (2.4%) patients. The AC on unclear side combined with the right PC involved in 1 (2.4%) patients (Fig. 3).

**Figure 4:**
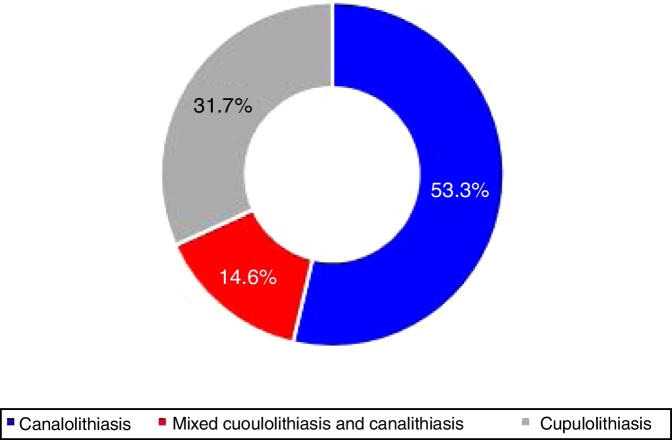
Distribution of MC-BPPV of the canalolithiasis and cupulolithiasis types.

According to the duration of nystagmus, e.g. <1 min and ≥1 min, 22 (53.7%) patients had canalolithiasis, 6 (14.6%) had cupulolithiasis, while 13 (31.7%) patients had mixed cupulolithiasis and canalithiasis (Fig. 4).

### Manual reduction

After initial manual reduction operations, 39.0% (14/41) patients with MC-BPPV were cured; manual reduction treatments were effective in 39.0% (16/41) patients, and not effective in 22.0% (9/41) patients. The total effective rate was 78.0% (32/41). The effective rates after treatment were 84.2% (16/19), 77.8% (7/9), 50.0% (1/2), and 72.8% (8/11), respectively in patients with PC-LC-BPPV, AC-LC-BPPV, bilateral PC-BPPV, and PC-AC-BPPV. There is no statistically significant difference in the treatment efficacy between patients with different types of MC-BPPV (*p* = 0.648, Table 3). During 1 week followup, 41.5% (17/41) patients with MC-BPPV were cured, manual reduction treatments were effective in 46.3% (19/41) patients, and not effective in 12.2% (5/41) patients, and the overall effective rate was 87.8% (36/41). The effective rates after 1-week treatment were 94.8% (18/19), 77.8% (7/9), 100% (2/2), and 81.8% (9/11) respectively, in patients with PC-LC-BPPV, AC-LC-BPPV, bilateral PC-BPPV, and PC-AC-BPPV, no statistically significant difference in the treatment efficacy was found between patients with different types of MC-BPPV (*p* = 0.541, Table 4).

**Table 3:** Effective rates after initial manual reduction in patients with MC-BPPV.

Types of MC-BPPV	*n*	Cure	Effective	Ineffective	Effective rate	*p*
PC-LC-BPPV	19	9 (47.4%)	7 (36.8%)	3 (15.8%)	84.2% (16/19)	0.648
AC-LC-BPPV	9	3 (33.3%)	4 (44.5%)	2 (22.2%)	77.8% (7/9)	
Bilateral PC-BPPV	2	0	1 (50.0%)	1 (50.0%)	50.0% (1/2)	
PC-AC-BPPV	11	4 (36.4%)	4 (36.4%)	3 (27.2%)	72.8% (8/11)	
Total	41	16 (39.0%)	16 (39.0%)	9 (22.0%)	78.0% (32/41)

**Table 4:** Effective rates after one week of manual reduction treatment in patients with MC-BPPV.

Types of MC-BPPV	*n*	Cure	Effective	Ineffective	Effective rate	*p*
PC-LC-BPPV	19	9 (47.4%)	9 (47.4%)	1 (5.2%0	94.8% (18/19)	0.174
AC-LC-BPPV	9	3 (33.3%)	4 (44.5%)	2 (22.2%)	77.8% (7/9)	
Bilateral PC-BPPV	2	1 (50.0%)	0	1 (50.0%)	1 (50.0%)	
PC-AC-BPPV	11	4 (36.3%)	5 (45.5%)	2 (18.2%)	81.8% (9/110)	
Total	41	17 (41.5%)	18 (43.9%)	6 (14.6%)	85.4% (35/41)	

**Figure 5:**
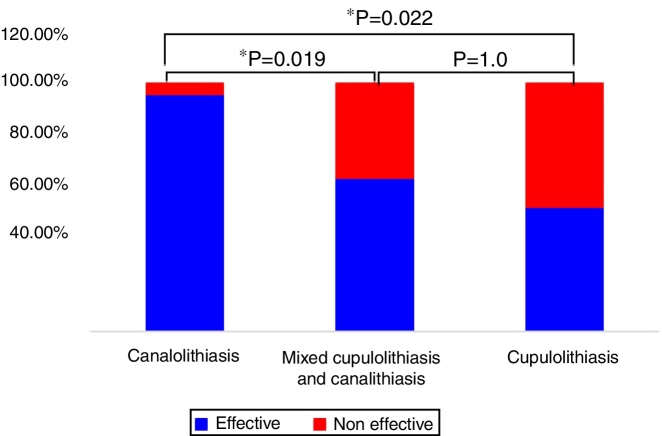
Comparison of the effective rates after initial manual reduction in patients with MC-BPPV-canalolithiasis and cupulolithiasis.

The effective rates after initial manual reduction were 95.50% (21/22), 61.50% (8/13), and 50.00% (3/6) respectively in MC-BPPV patients with canalolithiasis, mixed cupulolithiasis and canalithiasis, and cupulolithiasis. Manual reduction treatments were not effective in 4.50% (1/22), 38.50% (5/13), and 50.00% (3/6) MC-BPPV patients with canalolithiasis, mixed cupulolithiasis and canalithiasis, and cupulolithiasis, respectively. There were statistically significant differences in the treatment efficacy between MC-BPPV patients with canalolithiasis, and those with mixed cupulolithiasis and canalithiasis, and cupulolithiasis (*p* = 0.019, *p* = 0.022, respectively), while no statistically significant difference was found between MC-BPPV patients with mixed cupulolithiasis and canalithiasis, and cupulolithiasis (*p* = 1.000, Fig. 5).

The effective rate after 1 week's treatment were 95.50% (21/22), 76.9% (10/13) and 66.7 (4/6), respectively in MC-BPPV patients with canalolithiasis, mixed cupulolithiasis and canalithiasis, and cupulolithiasis. Manual reduction treatments were not effective in 4.50% (1/22), 23.1% (3/13), and 33.3% (2/6) MC-BPPV patients with canalolithiasis, mixed cupulolithiasis and canalithiasis, and cupulolithiasis, respectively. No statistically significant difference was found in the treatment efficacy between MC-BPPV patients with canalolithiasis, mixed cupulolithiasis and canalithiasis, and cupulolithiasis (*p* = 0.134, *p* = 0.107, *p* = 1.000, respectively).

## Discussion

### The incidence of MC-BPPV and the determination of affected side

In clinical practice, MC-BPPV is not uncommon in patients. In the present study, patients with MC-BPPV accounted for 10.5% (41/396) of all patients with BPPV visiting our hospital during the same period of time, which is consistent with previous reports demonstrating that the incidence of MC-BPPV was 6.8–20%.^16^, ^17^, ^18^, ^19^, ^20^, ^21^ In the present study, MC-BPPV was more common in older people above the age of 60 years, and with atherosclerotic risk factors (i.e., hypertension, hyperlipidemia, diabetes mellitus). It is speculated that these atherosclerotic risk factors can affect the blood supply to the inner ear, resulting in ischemia and hypoxia, which in turn may damage supporting cells in the macula utricule, causing abnormal metabolism and degeneration of otoliths; then the otoliths could be easily detached from the otolithic membrane, and could not be absorbed. In the present study, the male to female ratio is 1:1.7, which is basically consistent with the results of previous studies.^18^, ^19^, ^20^ We speculated that the decrease in the estrogen levels in middle-aged and older women may lead to calcium metabolism disorders in the body, which may affect otolith synthesis and function.^22^

MC-BPPV can affect different semicircular canals on one or both sides, and unilateral MC-BPPV is more common.^8^, ^21^, ^23^, ^24^ In the present study, among patients with unilateral MC-BPPV, the right side was more commonly affected, accounting for 67.7% of all cases, which was consistent with the results of previous studies.^19^, ^25^, ^26^ This may be related to the habit of sleeping in right lateral decubitus position. We also found that there was a high correlation between the side of Canal Paresis (CP) or hearing loss and the affected side of MC-BPPV (contingency coefficient = 0.602, *p* = 0.010). The concordance between the side of canal paresis (CP) or hearing loss and the side of MC-BPPV was further verified using Kappa statistic (*K*), high concordance was observed (*K* = 0.647, *p* = 0.004). At present, there are relatively few studies investigating the relationship between the side of reduced semicircular canal function and the affected side of BPPV. Wada et al.^27^ evaluated the inner ear function of PC-BPPV patients and revealed a high incidence of abnormal Canal Paresis (CP) on the affected side of PC-BPPV patients (*p* < 0.01), the recovery time in PC-BPPV patients with abnormal Canal Paresis (CP) was long after reduction treatment, and hearing loss also occurs more frequently on the affected side of BPPV (*p* < 0.01). Lin et al. found that the incidence of lateral semicircular canal dysfunction on the affected side in patients with BPPV was significantly higher than on the healthy side.^28^ Bi et al.^29^ found that the abnormal CP prevalence of BPPV was 57%, and the recurrent rate in BPPV patients with abnormal CP was significantly higher than those without abnormal CP. Therefore, we speculated that in patients with peripheral vestibular disorders or hearing impairment, if the affected side cannot be detected by nystagmus intensity, vestibular function evaluation can be used to help the determination of the affected side of BPPV, and patients can be treated with the appropriate manual reduction methods.

### Canal type distribution of MC-BPPV

In the present study, ipsilateral PC-LC-BPPV was the most common, accounting for 46.3% of all cases. At present, the results for the distribution of the involved canals of MC-BPPV from different studies are quite different. A previous study reported that the incidence of PC-LC-BPPV was 0.3–13.3%,^30^ while a multicenter study in Korea that included 1692 BPPV cases showed that MC-BPPV only accounted for 5% of BPPV cases, the proportion of PC-LC-BPPV among all patients having MC-BPPV in one ear was as high as 79.8%. Lopez-Escamez et al.^21^ and Shim et al.^18^ also showed that PC-LC-BPPV is the most common form of MC-BPPV. Other studies suggested that the most common form of MC-BPPV is bilateral PC-BPPV,^8^, ^20^, ^17^ which is often associated with damage to both ears caused by traumatic brain injury.^31^ But in the present study, the incidence of traumatic brain injury was low, since only 2 patients with MC-BPPV suffered traumatic brain injury, and they both had PC-BPPV; this may be the reason for the lower incidence of bilateral PC-BPPV. In addition, Steddin and Brandt^32^ showed that during DHT, non-standard procedures performed by the doctor may cause the movement of otolithic debris toward the ampullary end of the canal, resulting in inhibitory torsional nystagmus that beats toward the healthy side, so the unilateral PC-BPPV may be easily misdiagnosed as bilateral PC-BPPV, which is known as pseudo-bilateral PC-BPPV. Previous studies may have overestimated the incidence of unilateral PC-BPPV. Therefore, when we encounter patients with suspected bilateral PC-BPPV, further differential diagnosis is needed. The SHH test is helpful in the diagnosis of bilateral PC-BPPV. When the patient is held in the supine position with the head hanging straight, both PCs are simultaneously stimulated. If the nystagmus with torsional components has opposite direction and cancels each other out, and only the vertical up-beating nystagmus can be observed, the diagnosis of bilateral PC-BPPV is considered. If the torsional component is still relatively prominent, the possibility of bilateral PC-BPPV is not considered. In the 2 patients with bilateral PC-BPPV of our study, the SHH test induced vertical up-beating nystagmus in one case, and did not induce nystagmus in another case. And according to the intensity of nystagmus induced by bilateral D-HT, reduction was performed on the side with stronger nystagmus first, after performing bilateral D-HT again, and the vertigo and nystagmus did not completely disappear, which further supported the diagnosis of bilateral PC-BPPV.

In the present study, the incidence of MC-BPPV involving the AC was as high as 48.8%. However, previous studies reported that the incidence of AC-BPPV involving other semicircular canals is not high,^8^, ^18^ and the proportion of AC-BPPV involving other semicircular canals is not constant.^18^, ^19^, ^20^ The possible reasons may be as follows: (1) Our department is a higher-level vertigo center, and most patients in our center have transferred multiple times between different hospitals. Patients who had typical symptoms have been diagnosed in local hospitals, and many patients admitted to our center had no typical symptoms and signs with complex nystagmus. (2) For patients with MC-BPPV involving the AC, using diagnosis strategies, such as effectiveness of manual reduction treatment, the presence of canal conversion during reduction or follow-up period can help increase the diagnosis rate of AC-BPPV. The two major types of MC-BPPV involving the AC included AC-LC-BPPV and AC-PC-BPPV: (1) In the present study, AC-LC-BPPV accounted for 22.0% of all cases. During D-HT test, the nystagmus was usually down-beating on one or both sides, it could also be downbeating and torsional (apogeotropic), and very few patients showed torsional geotropic, downbeating nystagmus. It is speculated that during D-HT for MC-BPPV, multiple canals were simultaneously stimulated, the nystagmus induced by D-HT is the vector summation of each component of nystagmus.^21^ When D-HT is performed, the movement of otoliths in the LC induced apogeotropic, geotropic nystagmus, which may interfere with the torsional direction of the nystagmus induced by the movement of the otolith in the AC, resulting in nystagmus with torsional component beating toward the ground (geotropic). (2) AC-PC-BPPV accounted for 26.8% of all cases, the nystagmus was relatively complex, making diagnosis more difficult. Since the AC and PC are joined at one end to form the common crus at the lower part of the utricle, the otoliths are more likely to enter the common crus in the supine position. When turning from one side to the other, the otoliths may fall off simultaneously into the two semicircular canals, which cause the development of AC-PC- BPPV. The characteristics of the nystagmus are as follows: (a) For patients with AC-PC-BPPV in one ear (22.0%, 9/41), 8 patients showed up-beating nystagmus with geotropic torsional component on one side, and down-beating nystagmus on the other side during D-HT test, 1 patient showed up-beating nystagmus with geotropic torsional component, lasting for about 10 s, which was changed to down-beating nystagmus on one side, and down-beating nystagmus on the other side during D-HT test. The reason may be that when the otoliths are present in both the AC and PC, the otoliths can move simultaneously after performing D-HT on one side, so geotropic torsional up-beating nystagmus was first induced, which was then changed to down-beating nystagmus. Studies suggested that AC-BPPV should be treated first with manual reduction, and after treatment, vertical up-beating nystagmus with torsional component on one side induced by D-HT can help in the diagnosis of AC-PC-BPPV. After the second reduction treatment, the diagnosis of AC-PC-BPPV can be further confirmed if the treatment is effective.^33^, ^34^ (b) For patients with AC-PC-BPPV in both ears (2.4%, 1/41), D-HT on one side induced geotropic torsional with up-beating nystagmus, lasting for about 10 seconds, which was changed to down-beating nystagmus lasting ≥1 min, no nystagmus was observed on the other side on D-HT, and SHH induced down-beating nystagmus lasting >1 min. The possible reason may be that when D-HT is performed on one side, the ipsilateral PC and contralateral AC can both be stimulated, if otoconia is present in both the AC and PC, simultaneous stimulation of the two semicircular canals can produce up- and down-beating nystagmus with geotropic torsional. According to the nystagmus intensity, PC-BPPV was first treated with manual reduction, after treatment, D-HT performed on one side and SHH both induced down-beating nystagmus without torsional component. Then manual reduction was performed for AC-BPPV, and the treatment was effective. (c) For patients with AC-PC-BPPV with unclear affected side (2.4%, 1/41), bilateral D-HT and SHH can both induce down-beating nystagmus without torsional component and thus the affected side could not be determined. The study conducted by Casani et al.^35^ included 18 patients with AC-BPPV showed that the affected side in 12 patients could not be determined. For such patients, reduction treatment with Yacovino maneuver that does not require identification of the affected side should be performed. For complex MC-BPPV, dynamic positional nystagmus characteristics combined with therapeutic diagnosis can help improve diagnostic accuracy.

In addition, it is worth noting that LC-BPPV is mainly classified into three types. Usually, canalolithiasis of the posterior arm of the lateral canal is considered if geotropic horizontal nystagmus lasting <1 min is induced,^36^ and lateral semicircular canal cupulolithiasis is considered, if apogeotropic persistent horizontal nystagmus lasting more than 1 min is induced.^37^, ^38^ The canalolithiasis of the anterior arm of the lateral canal is considered if apogeotropic direction changing nystagmus lasting <1 min is induced.^39^ Canalolithiasis of the posterior arm of the lateral canal is the most common form of LC-BPPV. In the present study, MC-BPPV involving the LC was 56.1% and 14.3% (4/28) patients had canalolithiasis of the anterior arm of the lateral canal. Therefore, for patients with apogeotropic nystagmus, it is necessary to change the direction of nystagmus. The otoconia located in anterior arm of the lateral canal can move to the posterior arm with the change of body position, and then treatment for canalolithiasis of the posterior arm of the lateral canal can be performed.

In the present study, 46.3% patients with MC-BPPV had nystagmus lasting longer than 1 min, including14.6% patients with cupulolithiasis, 31.7% patients with mixed canalolithiasis and cupulolithiasis, and the initial manual reduction treatment was found to be more effective in patients with purely canalolithiasis than patients with mixed canalolithiasis and cupulolithiasis. Therefore, canalolithiasis and cupulolithiasis may co-exist simultaneously in patients with MC-BPPV; further investigation is need to confirm this findings.

### Manual reduction for MC-BPPV

At present, the order of manual reduction performed for MC-BPPV is still unclear. Parnes et al.^4^ suggested that manual reduction should be performed based on the intensity of symptoms, as well as the nystagmus induced by different canals, and more intense symptoms are induced by LC, so the order of reduction performed is LC, PC and AC. In the present study, PC-LC-BPPV is the most common type, and considering the problem of otolith displacement,^40^ if the Epley maneuver is used to treat PC-BPPV, the opening of common crus of the canals is higher than that of **t**he simple crus when patients returned to upright seated position at the end of the procedure, and otolith particles can easily enter the lateral semicircular canal under the action of gravity, causing LC-BPPV. Therefore, we performed reduction to treat PC-BPPV first and then LC-BPPV. AC-LC-BPPV and AC-PC-BPPV were also not uncommon in the present study, so in order to improve the accuracy of the diagnosis, we recommend treating the AC first. To reduce the otolith displacement caused by multiple reduction treatments performed, the two reductions in two canals should be performed with a time interval greater than 3 h.

Previous studies have shown that otolith repositioning maneuvers are effective in most of the patients with MC-BPPV.^8^, ^18^, ^19^ Soto-Varela et al.^19^ compared the effectiveness of repositioning maneuvers for the treatment of SC-BPPV and MC-BPPV: they found that more than 90% of patients in both groups were cured. In the present study, the effective rates after initial treatment and one-week treatment were 72.8% and 85.4% respectively, in patients with MC-BPPV, which was slightly lower than the findings of previous study. This occurs possibly because of the following reasons: (1) The conditions of patients with MC-BPPV are complex: 14.6% patients had cupulolithiasis, 31.7% patients had mixed canalolithiasis and cupulolithiasis. Those complex conditions can lead to poor reduction.^41^ And we found that repositioning maneuvers were more effective in patients with purely canalolithiasis compared to those with mixed canalolithiasis and cupulolithiasis, and purely cupulolithiasis. (2) Due to abnormal calcium metabolism in older people, the otolith particles are too large, which can narrow the semicircular canal, or increase the endolymph viscosity, and decrease endolymph fluid velocity with otoconial debris trapped in the semicircular canal; (3) MC-BPPV patients with old age, obesity, severe cervical spondylosis, and cardiovascular diseases showed reduced body flexibility and poor coordination during the reduction process. It is difficult for those patients to complete the reduction process according to the required speed and position, thereby reducing the success rate of reduction; (4) Dysfunction of a semicircular canal or utricle in older patients may cause a continuous dropping of otoliths into the semicircular canals.

### Study limitations

The limitation of the study is the small sample size; some results may not be statistically significant due to the small sample size. Further studies with a larger sample size are needed to confirm the results.

## Conclusion

Taken together, our results showed that (1) MC-BPPV was not uncommon in clinical practice, especially in patients older than 60 years old, and in patients who had comorbidities, such as hypertension, diabetes, osteoporosis; (2) PC-LC-BPPV was the most common type of MC-BPPV, MC-BPPV involving AC was also not uncommon, which was commonly attributed to canalithiasis; (3) The right side is more frequently involved in MC-BPPV, bithermal caloric tests and pure tone audiometry may help in the determination of the affected side; (4) Otolith repositioning maneuver was effective in most of the patients with MC-BPPV, which was more effective in treating patients with purely canalolithiasis compared to those with mixed canalolithiasis and cupulolithiasis, and purely cupulolithiasis after initial treatment.

## Conflicts of interest

The authors declare no conflicts of interest.
